# Targeting mitochondria in the aged cerebral vasculature with SS-31, a proteomic study of brain microvessels

**DOI:** 10.1007/s11357-023-00845-y

**Published:** 2023-07-17

**Authors:** Abigail Seman, Partha K. Chandra, Stephanie D. Byrum, Samuel G. Mackintosh, Allen J. Gies, David W. Busija, Ibolya Rutkai

**Affiliations:** 1 Department of Pharmacology, Tulane University School of Medicine, 1430 Tulane Avenue, New Orleans, LA 70112 USA; 2 Tulane Brain Institute, Tulane University, 200 Flower Hall, New Orleans, LA 70118 USA; 3 Department of Biochemistry and Molecular Biology, University of Arkansas for Medical Sciences, 4301 West Markham Street, Little Rock, AR 72205 USA

**Keywords:** Aging, Mitochondria, Brain microvasculature, Proteomics

## Abstract

Cognitive impairment and dementias during aging such as Alzheimer’s disease are linked to functional decline and structural alterations of the brain microvasculature. Although mechanisms leading to microvascular changes during aging are not clear, loss of mitochondria, and reduced efficiency of remaining mitochondria appear to play a major role. Pharmacological agents, such as SS-31, which target mitochondria have been shown to be effective during aging and diseases; however, the benefit to mitochondrial- and non-mitochondrial proteins in the brain microvasculature has not been examined. We tested whether attenuation of aging-associated changes in the brain microvascular proteome via targeting mitochondria represents a therapeutic option for the aging brain. We used aged male (> 18 months) C57Bl6/J mice treated with a mitochondria-targeted tetrapeptide, SS-31, or vehicle saline. Cerebral blood flow (CBF) was determined using laser speckle imaging during a 2-week treatment period. Then, isolated cortical microvessels (MVs) composed of end arterioles, capillaries, and venules were used for Orbitrap Eclipse Tribrid mass spectrometry. CBF was similar among the groups, whereas bioinformatic analysis revealed substantial differences in protein abundance of cortical MVs between SS-31 and vehicle. We identified 6267 proteins, of which 12% were mitochondria-associated. Of this 12%, 107 were significantly differentially expressed and were associated with oxidative phosphorylation, metabolism, the antioxidant defense system, or mitochondrial dynamics. Administration of SS-31 affected many non-mitochondrial proteins. Our findings suggest that mitochondria in the microvasculature represent a therapeutic target in the aging brain, and widespread changes in the proteome may underlie the rejuvenating actions of SS-31 in aging.

## Introduction

Aging has profound effects on vascular structure and function. Decreased vascular density, altered biomechanical properties, increased vascular tortuosity or increased cell permeability, and altered vasoreactivity contribute to an impaired neurovascular unit and reduced brain blood flow [1–5]. These aging-associated changes, which are exacerbated in AD or hypertension when coupled with regional vulnerabilities, might lead to brain hypoperfusion [6–8]. Emerging evidence supports the role of aging-associated vascular pathologies in dementias, including AD, specifying them as risk factors [9, 10]. These studies demonstrate that structural and functional integrity of the vasculature are essential in the maintenance of normal brain function. Mitochondria provide energy to the cerebral microvasculature for the maintenance of normal cellular functions, vasoreactivity, blood–brain barrier (BBB) maintenance and transport functions, protection against reactive oxygen species (ROS), and cellular repair and replacement [11, 12]. The high energy demand of the brain is maintained by mitochondria in vascular and non-vascular cells; however, age-related dysfunction of mitochondria leads to adverse changes in the brain microcirculation, with associated cognitive impairment and development of dementias such as AD [5, 13–15]. In support of this view, we showed that mitochondrial proteins decrease during aging in cerebral microvessels of mice, which was linked to a severe decrease in mitochondrial respiration [16, 17]. Surprisingly, we did not detect a compensatory increase in glycolysis during aging. Rather, aging decreased most protein levels involved in glycolysis leading to reduced energy production via this pathway. In addition, aging-associated increased oxidative stress, due in part to increased ROS production by dysfunctional mitochondria, in combination with an impaired antioxidant system, might exacerbate aging-associated vascular structure and dysfunction posing a risk for cerebrovascular and neurological pathologies. Thus, restoration of mitochondrial health in the brain microvasculature is important for the prevention of structural and functional impairment of the vasculature and neurological pathologies during aging. Specifically, a rejuvenated or maintained mitochondrial structure might facilitate an energetically efficient electron transport chain, leading to less ROS production and subsequently less oxidative stress and damage, and an improved cellular function. Although a number of mitochondrial-targeting agents have been found to be beneficial in disease states and aging, one of the most effective drug appears to be the SS-31 peptide (D-Arg-2′6′-dimethylTyr-Lys-Phe-NH2), also known as bendavia or elamipretide, which target cardiolipin in the mitochondria [18, 19]. However, it is unclear whether SS-31 effects are limited to the mitochondria or involve other proteins following improvement of mitochondrial function. To address this issue, we examined the effect of SS-31 peptide on the brain microvascular proteome of aged mice.

## Materials and methods

### Animals

Aged (18 months and older), male, C57Bl6/J mice were obtained (*n* = 10; #000664) from The Jackson Laboratory. Mice were housed in the vivarium at Tulane University, in accordance with Guidelines of the Institutional Animal Care and Use Committee of Tulane University and the National Institutes of Health Office of Laboratory Animal Welfare, with standard light and dark cycles and free access to food and water. Experiments were carried out according to the Animal Research: Reporting in Vivo Experiments (ARRIVE) guidelines. Mice were randomly assigned to either drug (SS-31) or vehicle (saline) groups. Mice in the treatment group received a daily i.p. injection of saline-dissolved and sterile filtered 10 mg/kg of SS-31 (#S9803, Selleck Chemicals LLC, Houston, TX) for 14 days. This dose was based on reported improvement of neurovascular coupling, spatial working memory, motor skill learning in old mice, compared with vehicle-treated animals [18]. In the vehicle group, mice were injected with saline to equal the total saline plus SS-31 dose administered to the experimental animals, based on animal weight, for 14 days.

### Cerebral blood flow measurement

**Fig. 1:**
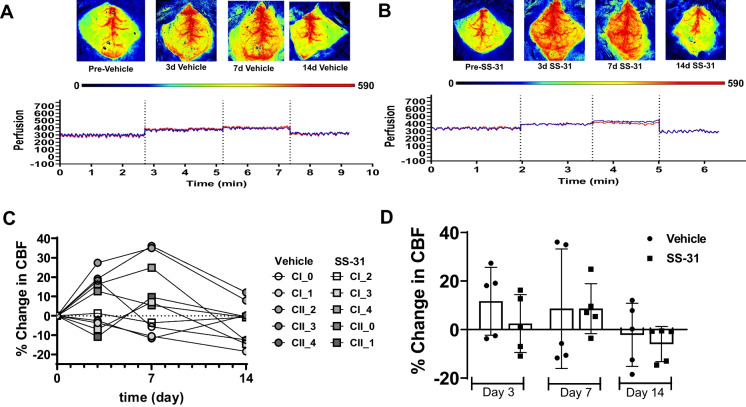
SS-31 does not affect baseline CBF. **A**–**B** Representative images and CBF recordings from vehicle (saline) and SS-31-treated animals, respectively. The perfusion intensity scale, below the images, indicates the level of brain perfusion. The deep blue color on the perfusion intensity scale denotes “0” or no perfusion, whereas red color corresponds to “590” or well-perfused brain areas. Blue and red lines of CBF recordings are indicative of CBF on the two hemispheres. **C** Shows the percent changes in CBF of individual animals, whereas panel **D** represents the summary of CBF changes in the groups, stratified by measurement days. Data expressed as mean ± SD; *n* = 5 mice in the SS-31 group and *n* = 5 mice in the vehicle-treated group

Cerebral blood flow (CBF) was measured using a Perimed PSIZ Laser speckle imager with PIMSoft 2.3.1. CBF was measured prior to treatment initiation on days 3, 7, and 14 (Fig. 1). Briefly, mice were anesthetized using 3% isoflurane in oxygen. Then, the head was shaved, and the skin was disinfected using alcohol and betadine. Afterward, mice were placed on a thermometer-controlled heating pad; eye ointment was used to prevent corneal dryness; and the head was secured on a stereotaxic frame. Prior to the 1-cm skin incision on the top of the head, animals received a 1 mg/kg s.c. ropivacaine injection (Ropivacaine HCl, 5 mg/mL, Somerset Therapeutics, Somerset, NJ). The skin was retracted; sterile imaging gel (Lubricating Jelly, MFR #16–8942, McKesson, Irving, TX) was applied on the skull and re-applied as needed during imaging. Anesthesia was maintained at 1.5% during CBF recording. After CBF recordings, the incision was closed using 7-mm wound clips, followed by application of betadine and a 1 mg/kg ropivacaine s.c. injection. Animals were allowed to recover in a clean cage, and then returned to their cage mates from both SS-31 and vehicle-treated groups. Additional fluid support (sterile saline) was provided for 3 days post-recording. CBF was recorded using a Perimed Pericam PSI Zoom Laser Speckle Imager and PIMSoft Software 2.3.1 using two different imaging settings. The following settings were used to record CBF: 12-cm distance, 1.6 cm × 1.6 cm, 25 images/s, and averaging 75 images/s, and 13-cm distance, 1 × 1 cm, 25 images/s, and averaging 25 images/s were used to capture high resolution images (Fig. 1A, B).

### MVs

After treatment, mice were transcardially perfused via cold PBS, and the brain was removed. Cortices were removed and homogenized, then cortical microvessels (MVs) were isolated via gradient centrifugation and size-filtering steps, as described by us [16, 20, 21] and others [17, 22]. The clarity of MV isolates (< 70 µm) composed of end arterioles, capillaries, and venules were monitored under light microscopy. Protein concentration of sonicated samples (in 1% SDS) was determined using BCA Protein assay [16, 20]. Then, samples were sent for proteomics and bioinformatics analysis to the University of Arkansas for Medical Sciences Proteomics and Bioinformatics core.

### Proteomics CME bHPLC TMT methods — Orbitrap Eclipse

Reduced, alkylated, and chloroform/methanol extraction purified MV samples were digested using sequencing grade trypsin (#V5111; Promega; Madison, WI). The tandem mass tag 10-plex isobaric labeled (#90110; Thermo; Waltham, MA) resulting proteins were combined into one sample group, then separated into 46 fractions on a 100 × 1.0 mm Acquity BEH C18 column (#186002346; Waters; Milford, MA) using an UltiMate 3000 UHPLC system (Thermo; Waltham, MA) with a 50-min gradient from 99:1 to 60:40 buffer A:B ratio under basic pH conditions, then consolidated into 18 super-fractions. Reverse phase XSelect CSH C18 2.5-μm resin (#186006729; Waters; Milford, MA) on an in-line 150 × 0.075-mm column using an UltiMate 3000 RSLCnano system (Thermo; Waltham, MA) was used to further separate each super-fraction. The eluted peptides were ionized by a 2.4 kV electrospray, and then subjected to mass spectrometric analysis on an Orbitrap Eclipse Tribrid mass spectrometer (Thermo; Waltham, MA) using multi-notch MS3 parameters. An FTMS analyzer in top-speed profile mode at resolution 120,000 over a range of 375 to 1500 m/z was used to obtain MS data. CID activation with normalized collision energy of 31.0 was followed by MS/MS data acquisition using the ion trap analyzer in centroid mode and normal mass range. Then, a synchronous precursor selection, up to 10 MS/MS precursors selected for HCD activation with normalized collision energy of 55.0, was followed by acquisition of MS3 reporter ion data using the FTMS analyzer in profile mode at a resolution of 50,000 over a range of 100–500 m/z. Buffer A, containing 0.1% formic acid and 0.5% acetonitrile, and Buffer B, containing 0.1% formic acid and 99.9% acetonitrile used for offline separation, were adjusted to pH 10 using ammonium hydroxide.

### Statistical analysis

#### CBF analysis

Elliptical, same-sized, regions of interest (ROI) were placed over the right and left hemispheres, with each ROI separated by a similar distance. Head alignment was done prior to recording, and if misaligned, ROIs were rotated accordingly. Mean perfusion was recorded, and CBF was expressed as percent change from the baseline for each hemisphere. Data distribution was tested in GraphPad Prism 9, using the Shapiro–Wilk test. Two-way-repeated measures ANOVA with Bonferroni’s multiple comparison test were used to compare the CBF values between groups. *p* < 0.05 was considered statistically significant. CBF data was plotted as % change in CBF of individual animals (Fig. 1C) as well as % change in CBF per treatment on the imaging days expressed as mean ± SD (Fig. 1D).

#### Proteomics analysis

**Fig. 2:**
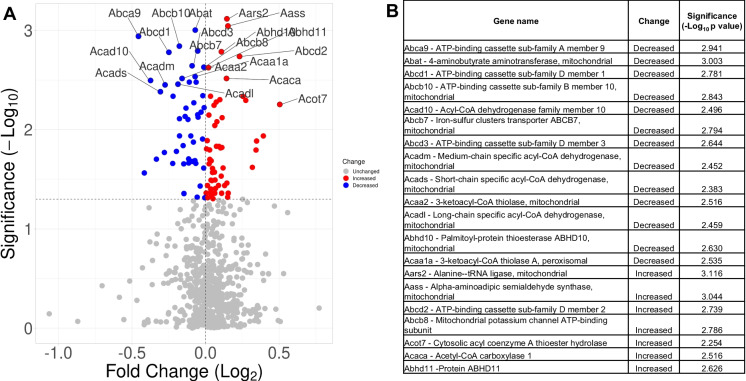
Effects of SS-31 on mitochondrial/mitochondria-related protein abundance in aged brain MVs. **A** 107 proteins, associated with mitochondria, were significantly differentially expressed in response to SS-31 treatment when compared with vehicle. **B** Table shows the top 20 significantly differentially expressed mitochondria-associated proteins, labeled in panel **A** using the mitochondrial/mitochondria-related data set

Proteomic data was analyzed by the UAMS Bioinformatics core, as described below. MaxQuant, quantitative proteomics software (Max Planck Institute of Biochemistry version 2.0.3.0) was used to analyze the mass spectrometry data with the following settings: parent ion tolerance of 3 ppm, fragment ion tolerance of 0.5 Da, reporter ion tolerance of 0.001 Da, trypsin enzyme with 2 missed cleavages, variable modifications including oxidation on M, acetyl on protein N-term, and fixed modification of Carbamidomethyl on C against the UniProt *Mus musculus* database (March 2021). Criteria for accepting identified proteins were set to less than 1.0% false discovery rate. Protein Prophet algorithm was used to assign protein probabilities [23]. ProteiNorm was used to quality check protein abundance data distribution via evaluating eight normalization methods [24], including log_2_ normalization (Log_2_), median normalization (Median), mean normalization (Mean), variance stabilizing normalization (VSN) [25], quantile normalization (Quantile) (Bolstad, B., *preprocessCore: A collection of pre-processing functions*. 2019), cyclic loess normalization (Cyclic Loess) [26], global robust linear regression normalization (RLR) [27], and global intensity normalization (Global Intensity) [27]. Total intensity pooled intragroup coefficient of variation (PCV), pooled intragroup median absolute deviation (PMAD), pooled intragroup estimate of variance (PEV), intragroup correlation, sample correlation heatmap (Pearson), and log_2_-ratio distributions were used to evaluate the individual performance of these methods, and the VSN normalization method was selected. Linear models for microarray data (limma) with empirical Bayes (eBayes) smoothing to the standard errors [26] was used for the normalized data statistical analysis. Proteins with a *p*-value < 0.05 were considered significant. Ensemble of gene set enrichment analyses (EGSEA), Bioconductor package, and Qiagen’s Ingenuity Pathway Analysis [28] were used to identify important protein networks and pathways based on the significant proteins. VolcaNoseR (Fig. 2A) was used to create volcano plots [29].

## Results

### CBF measurements

We used two-way-repeated measures ANOVA with Bonferroni’s multiple comparison test to analyze the data between groups stratified by days. Resting CBF levels were similar among the vehicle (Fig. 1A) and treatment groups (Fig. 1B). Percent changes in CBF of vehicle treated animals (*n* = 5) were 11.7 ± 13.9; 8.6 ± 24.7; − 2.1 ± 13.0 on days 3, 7, and 14, respectively (Fig. 1C). Interestingly, percent changes in CBF in response to treatment with SS-31 (*n* = 5) resulted in changes similar in magnitude to the vehicle group: 2.5 ± 11.9; 8.7 ± 10.3; − 5.9 ± 7.2 on days 3, 7, and 14, respectively (Fig. 1D).

### Proteomics

**Fig. 3:**
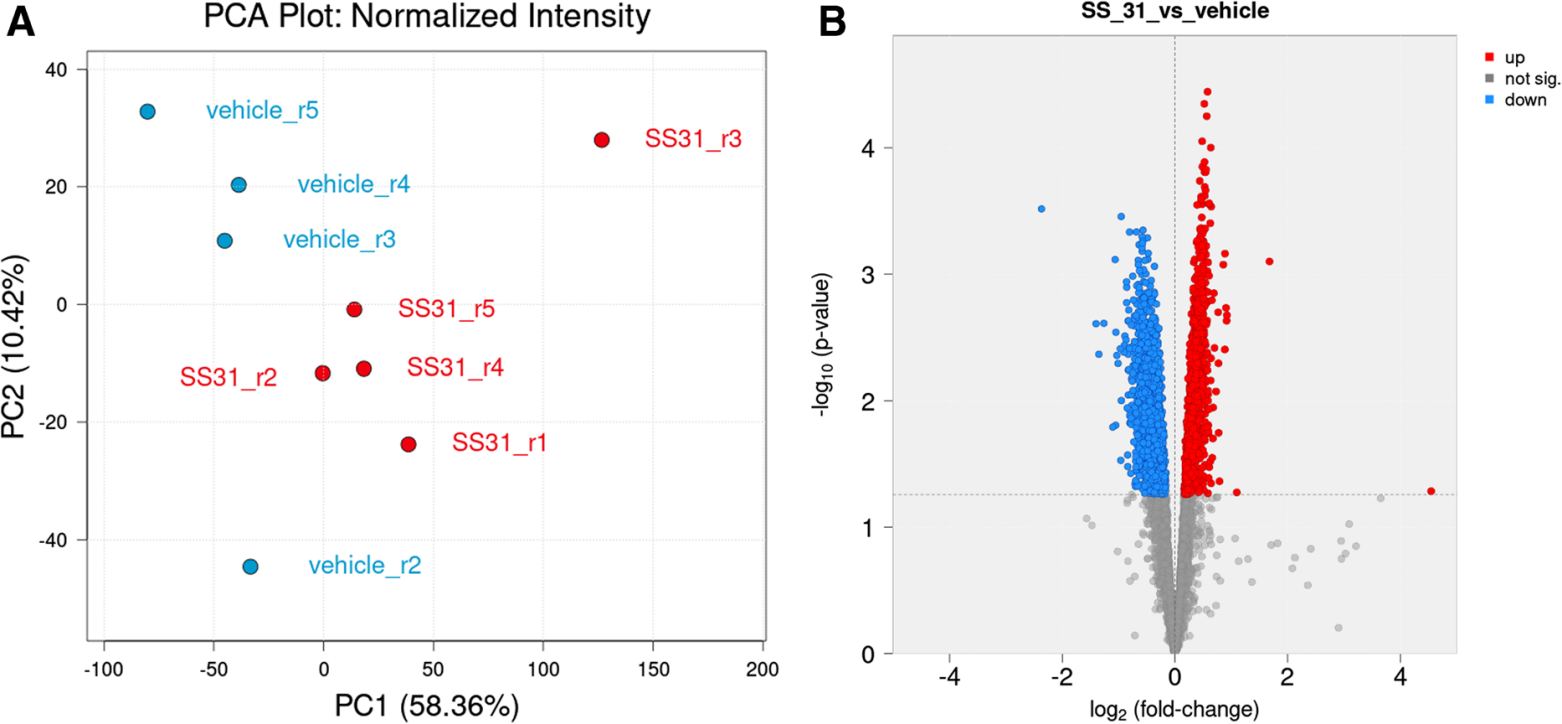
SS-31 treatment affects protein abundance of aged MVs. **A** Principal component analysis (PCA) plot shows protein expression profiles of brain MVs from SS-31 (red color) and vehicle (blue color)-treated aged mice. PC1 and PC2: Principal components 1 and 2, respectively. **B** Volcano plot shows significantly differentially expressed proteins between the SS-31 and vehicle groups in the global proteome of aged MVs. Red color indicates protein upregulation, blue color corresponds to protein downregulation, whereas gray denotes not significantly differentially expressed proteins in response to SS-31 treatments in isolated brain microvessels

**Fig. 4:**
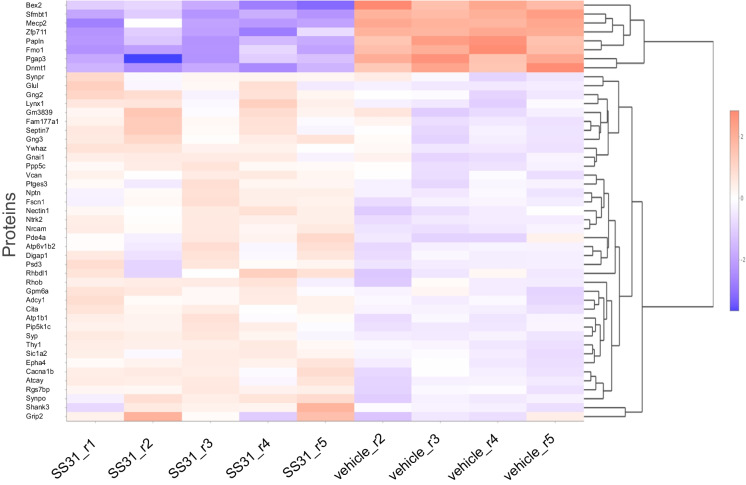
Heatmap of top 50 proteins in aged MVs. Heatmap shows the top 50 significantly differentially expressed proteins in aged MVs between the SS-31 and vehicle groups. *n* = 5 mice in the SS-31 group and *n* = 4 mice in the vehicle-treated group (1 vehicle was excluded from analysis)

**Fig. 5:**
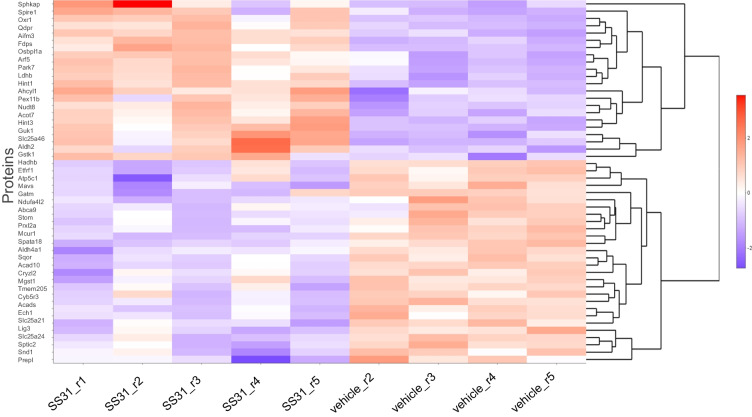
Heatmap of top 50 mitochondria-associated proteins in aged MVs. Heatmap shows the top 50 significantly differentially expressed mitochondria associated proteins in aged MVs between the SS-31 and vehicle groups. *n* = 5 mice in the SS-31 group and *n* = 4 mice in the vehicle treated group (1 vehicle was excluded from analysis)

Microvessel samples for analysis were obtained from individual mice. Sample sizes were: 5 for SS-31 and 5 for vehicle. One vehicle sample clustered with the treatment group (vehicle sample 1) and was excluded from statistical analysis using standard criteria. Principal component analysis (PCA) revealed that SS-31 and vehicle-treated groups separated 58.36% along PC1 and 10.42% along PC2 (Fig. 3A). Overall, we identified a total of 6267 proteins in our samples, of which 3263 were significantly differently expressed (*p* < 0.05) between the treatment and vehicle groups with non-adjusted *p* values (Fig. 3B; Fig. 4). Of these, 1837 remained significantly differently expressed (*p* < 0.05) when using the adjusted *p* value as screening criteria. Treatment with SS-31 resulted in the upregulation of 20 (of which three with *p* < 0.05) and downregulation of 13 proteins (of which 10 with *p* < 0.05) with fold change threshold between 1 and 4.5 and negative one and negative 2.4. We found 747 entries in our data set when filtered for mitochondrial proteins and those associated with mitochondria, using the MitoCarta3.0 [30] as a basis for comparison as well as the criteria of “mitochondria” in the protein name. Of 107 proteins that were significantly differentially expressed, 48 were downregulated, and 59 were upregulated in response to SS-31 treatment (Figs. 2A and 5).

The five top canonical pathways associated with our global data set included the synaptogenesis signaling pathway, spliceosomal cycle, androgen signaling, Huntington’s disease signaling, and 14–3-3-mediated signaling (Table 1), while our mitochondrial only data set was linked to mitochondrial dysfunction, sirtuin signaling pathway, oxidative phosphorylation, xenobiotic metabolism AhR signaling pathway, and geranylgeranyl diphosphate biosynthesis I (via mevalonate) (Table 1). The top upstream regulators associated with the overall data set included microtubule associated protein tau (MAPT; *p* = 1.33 × 10^−43^), cystatin D (CST5; *p* = 5.47 × 10^−34^), amyloid beta precursor protein (APP; *p* = 1.11 × 10^−27^), matrix metallopeptidase 3 (MMP3; *p* = 5.58 × 10^−24^), protein tyrosine phosphatase 4A1 (PTP4A1; *p* = 1.30 × 10^−20^), whereas the following upstream regulators were associated with the mitochondria only data set: TEA domain transcription factor 1 (TEAD1; *p* = 1.27 × 10^−18^), ATP synthase inhibitory factor subunit 1 (ATP5IF1; *p* = 1.53 × 10^−16^), caseinolytic mitochondrial matrix peptidase proteolytic subunit (CLPP; *p* = 1.17 × 10^−15^), tumor protein 53 (TP53; *p* = 5.00 × 10^−11^), and peroxisome proliferator-activated receptor alpha (PPARA; *p* = 7.84 × 10^−11^). In addition, IPA analysis of the top molecular and cellular functions associated with our overall data set included RNA post-transcriptional modifications, cellular assembly, organization, function, maintenance, development, growth, and proliferation, whereas energy production, lipid metabolism, small molecule biochemistry, molecular transport, and nucleic acid metabolism were associated with the mitochondria data set only.

**Table 1:** Top five canonical pathways

	Pathway name	*p* value	Overlap
Overall data	Synaptogenesis signaling pathway	1.12E − 36	41.6% 132/317
	Spliceosomal cycle	1.91E − 24	77.6% 38/49
	Androgen signaling	3.49E − 24	45.0% 76/169
	Huntington’s disease signaling	5.89E − 20	34.3% 97/283
	14–3-3-mediated signaling	1.91E − 18	44.9% 57/127
Mitochondrial data set	Mitochondrial dysfunction	1.34E − 15	8.8% 15/171
	Sirtuin signaling pathway	3.46E − 12	5.1% 15/292
	Oxidative phosphorylation	6.90E − 11	9.0% 10/111
	Xenobiotic metabolism AHR signaling pathway	4.36E − 05	5.7% 5/87
	Geranylgeranyl diphosphate biosynthesis I (via mevalonate)	6.63E − 05	16.7% 3/18

Using the MitoPathways3.0.gms, the significantly differentially expressed mitochondrial proteins were shown to be associated with different mitochondrial pathways (Fig. 2B).

#### OXPHOS

**Fig. 6:**
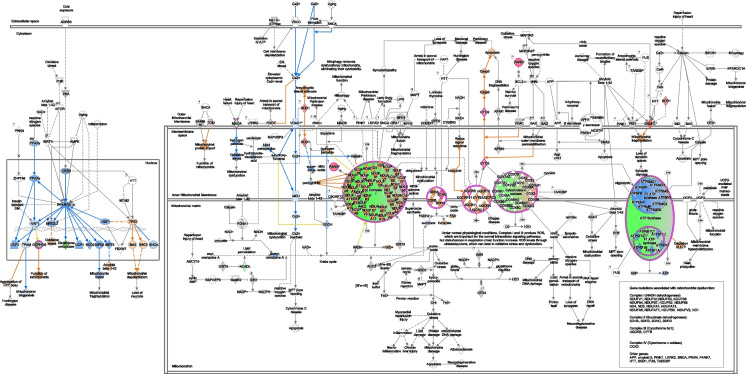
Mitochondrial dysfunction pathway. QIAGEN Ingenuity Pathway Analysis shows differentially expressed genes in our mitochondria only data set from SS-31 and vehicle-treated isolated brain microvessels from male, aged C57Bl/6J mice that are associated with mitochondrial dysfunction and oxidative phosphorylation canonical pathways

**Fig. 7:**
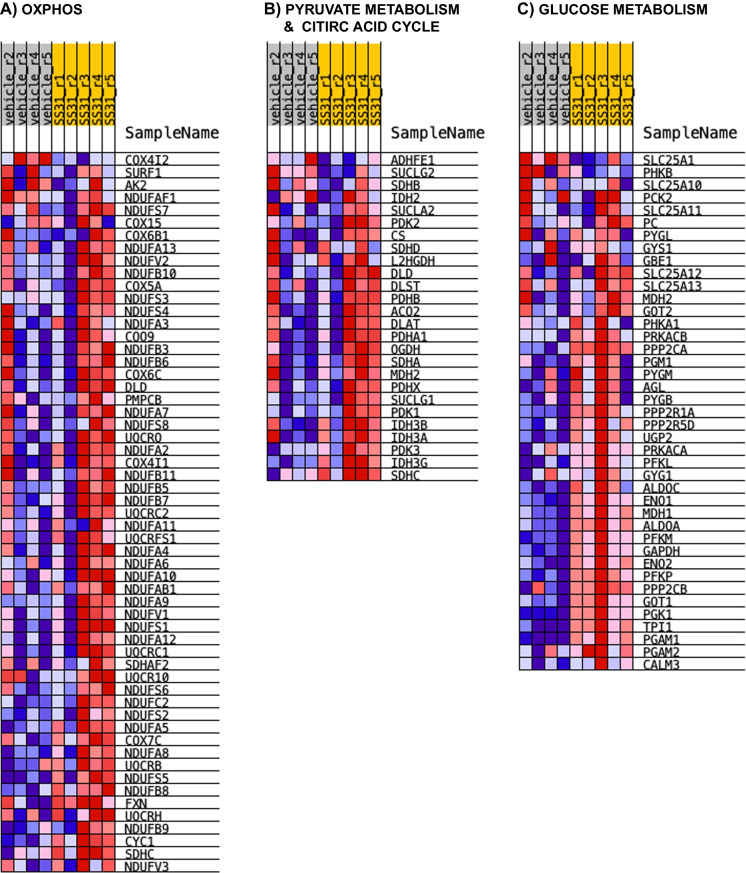
Gene Enrichment Set Analysis (GESA) heatmaps. SS-31 resulted in the enrichment of several proteins associated with **A** biological processes (GO_ OXIDATIVE_PHOSPHORYLATION) and **B** pyruvate metabolism and citric acid cycle (Reactome) within the mitochondrial data set, and in those identified in **C** glucose metabolism within the global data set

We identified 13 significantly differentially expressed proteins in our data set that are associated with OXPHOS using the MitoPathways table (13/168). Specifically, Complex I-associated proteins included the NADH dehydrogenase [ubiquinone] 1 alpha subcomplex subunit 5 (Ndufa5; SS-31 > vehicle), NADH dehydrogenase [ubiquinone] 1 alpha subcomplex subunit 8 (Ndufa8; SS-31 > vehicle), NADH dehydrogenase [ubiquinone] 1 beta subcomplex subunit 8 (Ndufb8; SS-31 > vehicle), and Complex I–15 kDa (Ndufs5; SS-31 > vehicle). Succinate dehydrogenase cytochrome b560 subunit (Sdhc; SS-31 > vehicle) was the only Complex II-associated protein that was affected by SS-31 treatment. Complex III-related subunit and assembly factor proteins included the cytochrome C1, heme protein (Cyc1; SS-31 > vehicle), ubiquinol-cytochrome c reductase binding protein (Uqcrb; SS-31 > vehicle), and BCS1 homolog, ubiquinol-cytochrome c reductase complex chaperone (Bcs1l; SS-31 > vehicle). Complex IV-affiliated subunit proteins and assembly factor included cytochrome c oxidase subunit 4I2 (Cox4i2; SS-31 < vehicle), cytochrome c oxidase subunit 7A-related protein (Cox7a2l; SS-31 > vehicle), and HIG1 hypoxia inducible domain family member 1A (Higd1a; SS-31 > vehicle). Only one Complex-related subunit, the ATP synthase F1 subunit gamma (Atp5c1; SS-31 < vehicle) was significantly differentially expressed. In addition, Cox7a2l and Higd1a are related to respirasome assembly according to the MitoPathways3.0.gms source, whereas cytochrome C, somatic (Cycs; SS-31 > vehicle) is associated with cytochrome C complex **(**Figs. 6 and 7A**)**. In addition, we found that SS-31 increased levels of proteins associated with macromolecules and complex assembly (data not shown).

#### Metabolism

Our analysis revealed 59 significantly differentially expressed proteins out of the 464 that were associated with different metabolic pathways. These significantly differentially expressed proteins were associated with distinct and/or multiple pathways (only some discussed here). Carbohydrate metabolism-associated proteins included the ATP citrate lyase (Acly; SS-31 > vehicle), hydroxyacyl-CoA dehydrogenase trifunctional multienzyme complex subunit beta (Hadhb; SS-31 < vehicle), and isocitrate dehydrogenase [NAD( +)] 3 non-catalytic subunit gamma (Idh3g; SS-31 > vehicle). Solute carrier family 25 member 1 or Tricarboxylate transport protein, mitochondrial (Slc25a1; SS-31 < vehicle) is associated with gluconeogenesis, whereas Idh3g, Acly, Slc25a1 as well as the Sdhc (SS-31 > vehicle) are associated with the TCA cycle. Glycolysis-associated proteins significantly increased in response to SS-31 included hexokinase (Hk1), glucose-6-phosphate isomerase (Gpi), ATP-dependent 6-phosphofructokinase (Pfkp), fructose-bisphosphate aldolase A/C (Aldoa/c), triosephosphate isomerase (Tpi), glyceraldehyde-3-phosphate dehydrogenase (GAPDH), phosphoglycerate kinase 1 (Pgk1), phosphoglycerate mutase 1 (Pgam1), alpha and gamma enolase (Eno1/2), and pyruvate kinase (Pkm). Fatty acid oxidation-related proteins included the acyl-CoA dehydrogenase family member 10 (Acad10; SS-31 < vehicle), acyl-CoA dehydrogenase long chain (Acadl; SS-31 < vehicle), acyl-CoA dehydrogenase medium chain (Acadm; SS-31 < vehicle), acyl-CoA dehydrogenase short chain (Acads; SS-31 < vehicle), acyl-CoA dehydrogenase very long chain (Acadvl; SS-31 < vehicle), acyl-CoA thioesterase 11 (Acot11; SS-31 > vehicle), carnitine palmitoyltransferase 1A (Cpt1a; SS-31 < vehicle), 2,4-dienoyl-CoA reductase 1 (Decr1; SS-31 < vehicle), hydroxyacyl-CoA dehydrogenase trifunctional multienzyme complex subunits alpha (Hadha; SS-31 < vehicle; also associated with cardiolipin synthesis) and beta (Hadhb; SS-31 < vehicle). Oxysterol binding protein like 1A (Osbpl1a; SS-31 > vehicle) is associated with cholesterol, bile acid, steroid synthesis pathway as well as phospholipid metabolism along with peroxiredoxin 6 (Prdx6; SS-31 > vehicle) and serine palmitoyltransferase long chain base subunit 2 (Sptlc2; SS-31 < vehicle). Amino acid metabolism pathway-related proteins include aldehyde dehydrogenase 4 family member A1 (Aldh4a1; SS-31 < vehicle), glutaminase (Gls.1; SS-31 > vehicle), glyoxylate and hydroxypyruvate reductase (Grhpr; SS-31 > vehicle), lactate dehydrogenase B (Ldhb; SS-31 > vehicle), monoamine oxidase A (Maoa; SS-31 < vehicle), as well as the previously mentioned Hadha, Hadhb, and Slc25a21; (Fig. 7B, C). Our findings indicate that aging progressively affects most of the major energy producing pathways, including glycolysis and OXPHOS possibly compromising ATP production. In addition, increase in protein abundance indicates the ability of SS-31 to ramp up protein synthesis related to the homeostasis of major fuel sources; however, further studies are needed to establish the beneficial effects of SS-31 on the function of these proteins.

#### Mitochondrial dynamics and surveillance

Nine, significantly differentially expressed proteins of 102 were associated with mitochondrial quality control processes. Specifically, fission-associated proteins included the dynamin 1 like (Dnm1l.1 and Dnm1l.2; SS-31 > vehicle), spire type actin nucleation factor 1 (Spire1; SS-31 > vehicle), and Slc25a46. Adenosylhomocysteinase-like 1 (Ahcyl1; SS-31 > vehicle) and spire1 are also related to organelle contact site, whereas metaxin 2 (Mtx2; SS-31 < vehicle) and Slc25a46 indicated involvement in intramitochondrial membrane interactions. Parkinson disease protein 7 homolog (Park7; SS-31 > vehicle) and PGAM family member 5, and mitochondrial serine/threonine protein phosphatase (Pgam5; SS-31 > vehicle) were listed as mitophagy/autophagy related proteins, while Aifm3 (SS-31 > vehicle) and Cycs (SS-31 > vehicle) were associated with apoptosis.

#### Small molecule transport

We found that 10 of 85 proteins were also related to transport mechanisms, which included ATP binding cassette subfamily A member 9 (Abca9; SS-31 < vehicle), ATP binding cassette subfamily A member (Abcb10; SS-31 < vehicle), ATP binding cassette subfamily D members 1 (Abcd1; SS-31 < vehicle) and 2 (Abcd2; SS-31 > vehicle), sideroflexin 4 (Sfxn4; SS-31 > vehicle), mitochondrial 2-oxodicarboxylate carrier (Slc25a21; SS-31 < vehicle), calcium-binding mitochondrial carrier protein SCaMC-1 (Slc25a24; SS-31 < vehicle), mitochondrial coenzyme A transporter (Slc25a42; SS-31 > vehicle), and solute carrier family 25 member 46 (Slc25a46; SS-31 > vehicle).

#### Detoxification

We found that 15 of 51 proteins were significantly differentially expressed. Specifically, 11 of 27 ROS and glutathione metabolism-associated proteins were significantly different. These included hydroxyacylglutathione hydrolase (Hagh; SS-31 > vehicle), microsomal glutathione s-transferase 1 (Mgst1; SS-31 < vehicle), methionine sulfoxide reductase A (Msra; SS-31 > vehicle), nitrilase 1 (Nit1; SS-31 > vehicle), oxidation resistance 1 (Oxr1; SS-31 > vehicle), peroxiredoxin 2 (Prdx2; SS-31 > vehicle), peroxiredoxin 5 (Prdx5; SS-31 > vehicle), peroxiredoxin 6 (Prdx6; SS-31 > vehicle), peroxiredoxin like 2A (Prxl2a; SS-31 < vehicle), superoxide dismutase 1 (Sod1; SS-31 > vehicle), and thioredoxin reductase 1 (Txnrd1; SS-31 > vehicle). These findings indicate that SS-31 exerts its antioxidant effects in the brain MVs, possibly contributing to a decreased ROS production and a subsequently decreased oxidative stress.

#### Metals and cofactors

We identified 11 significantly differentially expressed proteins out of 120 associated with Cpt1a (SS-31 < vehicle): coenzyme A metabolism (Nudix Hydrolase 8 [Nudt8], SS-31 > vehicle; pantothenate kinase 2 [Pank2], SS-31 > vehicle; solute carrier family 25 member 42 [Slc25a42], SS-31 > vehicle); heme synthesis and processing (ATP binding cassette subfamily b member 10 [Abcb10], SS-31 < vehicle; and protoporphyrinogen oxidase [Ppox], SS-31 < vehicle); tetrahydrobiopterin and vitamin metabolism pathways quinoid dihydropteridine reductase (Qdpr; SS-31 > vehicle); Fe-S, and heme-containing proteins including the apoptosis inducing factor mitochondria-associated 3 (Aifm3; SS-31 > vehicle), Cyc1 (SS-31 > vehicle), and Sdhc (SS-31 > vehicle).

## Discussion

The major finding of our study is that targeting mitochondria using SS-31 resulted in widespread beneficial changes in the brain microvascular proteome beyond those involving mitochondria in aged male mice. These changes may identify mechanisms underlying the beneficial effects of SS-31 on cerebrovascular function, neurovascular coupling, and cognitive function during aging and other conditions [18, 31]. We speculate that SS-31 mediated, enhanced mitochondrial function, and reduced ROS as well as improved glycolysis may lead to improved structural and functional status of brain MVs.

Aging is associated with a decline in cognitive function and cerebral blood flow, observed in both animal and epidemiological [reviewed in [4, 5]] studies [3, 9, 10, 32, 33]. The beneficial effects of SS-31 on CBF and neurovascular coupling were first demonstrated in aged animals by Tarantini et al. [18] CBF responses of treated old mice to whisker stimulation were improved and comparable with whisker stimulation responses elicited in young mice [18]. In our experiments, laser speckle contrast imaging showed similar CBF between treated and vehicle groups, most likely due to the lack of whisker stimulation or pharmacological challenge. In addition, baseline CBF was similar after experimental stroke in young mice between the bendavia (SS-31) and vehicle-treated groups [34]. These suggest that protein abundance changes in our experiments were due to SS-31 treatment rather than to improved CBF. Interestingly, Tarantini’s SS-31 treatment did not affect myogenic tone or smooth muscle vasodilatory response to sodium nitroprusside in isolated middle cerebral arteries; however, SS-31 improved endothelial-dependent vasodilation in response to acetylcholine and ATP [18]. These findings indicate rescued neurovascular coupling and improved endothelial function in the treated aged group, compared with aged-matched controls. Tarantini et al. further investigated the role of SS-31 treatment in nitric oxide (NO)-mediated signaling and reported no change of Nos1 (nNOS) and Nos3 (eNOS) mRNA [18]. Similarly, we detected no differences in protein abundance of nNOS and eNOS; however, SS-31 significantly decreased Nos2 (iNOS) expression in isolated aged brain microvessels. Another beneficial effect, attributed to SS-31, is its ability to decrease oxidative stress, which is demonstrated in the cerebral vasculature of male hypertensive SHR rats exposed to mild traumatic brain injury (mTBI) [31]. Both studies explored the role of SS-31 in attenuating/altering NOX signaling, which is increased in aging and in hypertension, facilitating a greater oxidative stress. Due to the technical limitation of proteomics, we could not detect these proteins in our samples. We identified, however, several proteins involved in ion channel activities such as Ca^2+^ signaling or the endothelin-conversion pathway, which were significantly different between the treated group and vehicle. Thus, the protective effects of SS-31 on the brain vasculature warrant further investigation beyond the NO and NADPH oxidase pathways. Similarly, SS-31 improved the aging phenotype in different organs and tissues. Treatment with elamipretide (SS-31) for 8 weeks restored reduced diastolic function in treated old mice, whereas it had no effect in treated young mice [35]. Furthermore, the beneficial effects of SS-31/elamipretide on skeletal muscle function [36, 37] and in angiotensin II-induced cardiomyopathy [38] were reported by different groups.

Several laboratories investigated the basis of SS-31-mediated functional improvement in aged animals via assessing mitochondrial ROS production and mitochondrial respiration. In accordance with functional studies, the positive pleiotropic effects of SS-31 [19] such as decreased oxidative stress in old cardiomyocytes [39] and in angiotensin-stimulated neonatal cardiomyocytes [38], or improved mitochondrial respiration and ATP production [18, 40] were reported by independent groups; however, SS-31 had no significant effect in young animals [40] or young cells. In addition, Zhang et al. (2020) found that SS-31 interacts with ANT1, a protein that was not affected by SS-31 in our study and also observed a decreased ROS production in old, SS-31-treated cardiomyocytes; however, the exact mechanism was not explored, and whether it was due to the interaction between SS-31 and ANT1 or to an improved membrane structure via SS-31 needs further investigation. The antioxidant properties of SS-31/elamipretide is thought to contribute to its beneficial effects in the brain. SS-31 is reported to decrease mitochondrial ROS production in old primary cerebromicrovascular endothelial cells without affecting the antioxidant superoxide dismutase 1 (Sod1) and 2 (Sod2) levels in brain samples of treated old mice [18]. We found that SS-31 significantly increased Sod1 but not Sod2 expression in aged brain microvessels. Likewise, SS-31 resulted in decreased ROS production in middle cerebral arteries from SHR rats exposed to mTBI [31], in the hippocampus of aged mice [41], or in tert-butyl hydroperoxide treated N2A cells [42]. In support of the antioxidant effects of SS-31, we identified several significantly differentially expressed proteins associated with the detoxification/antioxidant defense system. Some of these differentially expressed proteins play a role in glutathione and D-lactic acid production (Hagh), in reversible oxidation–reduction of methionine sulfoxide (Msra), in hydrolysis of deaminated glutathione (Nit1), in the reduction of hydrogen peroxide (Prdx2/5/6) or disulfideprotein thioredoxin (Txnrd1), and in the protection from oxidative damage (Sod1, Oxr1).

Among the above-mentioned beneficial effects of SS-31, we found energy producing pathways were also affected in treated aged samples. We showed reductions in mitochondrial proteins composing Complexes I–IV in the microvasculature of aged mice [16]. Our findings were independently confirmed by studies showing reduced mitochondrial respiration in aged mice [17]. Increased glycolysis has been suggested to maintain energy production in the face of mitochondrial dysfunction; however, studies by us and others on energy production via glycolysis show that glycolysis was also impaired in aged brain MVs [16, 43, 44]. GSEA analysis of the global proteome revealed that proteins associated with glucose metabolism, including glycolysis and gluconeogenesis were upregulated in the treatment group, compared with vehicle. Thus, we postulate that energy failure in the brain microvasculature causes structural damage, BBB disruption, and impaired neurovascular coupling leading to cognitive impairment, susceptibility to strokes, and induction of dementias such as AD. On the other hand, SS-31 resulted in improved neurovascular coupling and subsequently enhanced cognitive function in treated old mice [18]. In addition, SS-31 increased ATP citrate lyase (ACLY; *p* < 0.05), an enzyme supporting lipid biosynthesis and protein abundance of enzymes involved in alpha-ketoglutarate production (Idh3g, *p* < 0.05; Idh3a and Idh2b). We did not assess enzyme activities; however, an increase in abundance may suggest an elevation of acetyl-CoA or the key TCA metabolite. Furthermore, SS-31 treatment increased the abundance of proteins involved in Complex I and I-associated electron transfer as well as three proteins associated with the ubiquinol-cytochrome c oxidoreductase complex. Upregulation of proteins associated with the cytochrome c oxidase and Complex V may result in a more efficient electron transfer, contributing to a decreased ROS production. This finding is in accordance with improved mitochondrial respiration in treated, cultured, aged, and cerebromicrovascular endothelial cells [18].

There were a few limitations to our study. We only used extracted MVs and examined the proteome after 14 days of SS-31 treatment. Thus, we could not determine the time course of SS-31 changes on the microvasculature. However, we used this protocol based on previous studies, which showed protection of neurovascular coupling in aged animals. At the time of the study, we could not separate the different microcirculation cell types; however, we are exploring methods that will allow this to yield sufficient cell-specific proteins for proteomic analysis. We used male mice because our proteomic examinations [16] showed only modest sex-dependent differences in aged mice.

In conclusion, our results support and extend studies reporting on the ability of SS-31 to cross the blood–brain barrier, improve or restore cerebral vascular function, and remediate dysfunction of cerebral vascular mitochondria in aged mice. These results warrant further investigation of the therapeutic potential of mitochondria in different disease models, with the inclusion of both sexes as well as on the effects of SS-31 on mitochondrial structure and function using intra- and extracranial vessels. Our results further confirm the multifaceted effects of SS-31 on the brain vasculature, requiring the consideration of possible interplay among transcriptional, translational, and post-translational modifications, as well as enzyme activities that might contribute to its beneficial effects. In addition, future studies are needed to determine the time course of effectiveness of SS-31, as well as cellular and vascular bed-specific similarities and differences in the brain.

## Data Availability

The data associated with this study available from the corresponding author upon reasonable request. The mass spectrometry proteomics data have been deposited to the ProteomeXchange Consortium via the PRIDE partner repository with the dataset identifier PXD038483 and 10.6019/PXD038483.
